# DOES REHABILITATION IMPROVE WORK PARTICIPATION IN PATIENTS WITH CHRONIC SPINAL PAIN AFTER SPINAL SURGERY: A SYSTEMATIC REVIEW

**DOI:** 10.2340/jrm.v57.25156

**Published:** 2025-01-03

**Authors:** Jonas CALLENS, Olivia LAVREYSEN, Lisa GOUDMAN, Ann DE SMEDT, Koen PUTMAN, Dominique VAN DE VELDE, Lode GODDERIS, Dries CEULEMANS, Maarten MOENS

**Affiliations:** 1 STIMULUS research group, Vrije Universiteit Brussel, Jette; 2 Cluster Neurosciences, Center for Neurosciences (C4N), Vrije Universiteit Brussel, Brussels; 3 Interuniversity Centre for Health Economics Research (I-CHER), Department of Public Health (GEWE), Faculty of Medicine and Pharmacy, Vrije Universiteit Brussel, Jette; 4 Centre for Environment and Health, Department of Public Health and Primary Care, KU Leuven (University of Leuven), Leuven; 5 Pain in Motion Research Group (PAIN), Department of Physiotherapy, Human Physiology and Anatomy, Faculty of Physical Education & Physiotherapy, Vrije Universiteit Brussel, Jette; 6 Department of Neurosurgery, Universitair Ziekenhuis Brussel, Jette; 7 Center for Neurosciences (C4N), Vrije Universiteit Brussel, Jette; 8 Research Foundation Flanders (FWO), Brussels; 9 Department of Physical Medicine and Rehabilitation, Universitair Ziekenhuis Brussel, Jette; 10 Faculty of Medicine and Healthcare Sciences, Department of Rehabilitation Sciences, Occupational Therapy Program, Ghent University, Ghent; 11 IDEWE, External Service for Prevention and Protection at Work, Heverlee; 12 Department of Radiology, Universitair Ziekenhuis Brussel, Jette, Belgium

**Keywords:** chronic pain, failed back surgery syndrome, rehabilitation, return to work, systematic review

## Abstract

**Objective:**

Patients with therapy-refractory chronic spinal pain after spinal surgery experience increased disability, resulting in substantial loss of employment and consequently lower quality of life. Despite findings that rehabilitation improves socio-economic outcomes in other chronic pain conditions, evidence for patients with chronic spinal pain after spinal surgery is limited. A systematic review was conducted to provide an overview of rehabilitation interventions and their effectiveness to improve work participation for patients with chronic spinal pain after spinal surgery.

**Methods:**

MEDLINE (via PubMed), Scopus, Embase, and Web of Science, were systematically searched. Risk of bias was assessed using the modified Downs and Black checklist and GRADE was used to assess certainty of evidence. The review protocol was prospectively registered on PROSPERO (CRD42022346091).

**Results:**

The search yielded 1,289 publications. Full-text screening of 48 articles resulted in the inclusion of 6 publications. The included interventions comprised multiple treatment components, consisting of back school, self-care, functional restoration, multidisciplinary rehabilitation, physiotherapy, and digital care programmes to improve work participation.

**Conclusion:**

Rehabilitation to improve return to work for patients with chronic spinal pain after spinal surgery was supported only by low-certainty evidence. Rehabilitation therapies that are personalized and that integrate the patient’s work seem most suitable.

## LAY ABSTRACT

Patients with chronic pain after previous spinal surgery experience significant disability, pain, and loss of employment. Return to work is an important treatment goal for these patients; however, there is no clear overview of which rehabilitation components can reinforce work resumption. We conducted a systematic literature review to provide an overview of the content of rehabilitation and its effectiveness. A broad variety of treatment components were revealed, all with a specific focus on work participation. Moreover, all programmes consisted of an interplay between different disciplines. Rehabilitation therapies that are personalized and that integrate the patient’s work seem most suitable.

Globally, low back pain and neck pain are consistently presented among the top 4 leading causes of years lived with disability (1, 2). Both conditions significantly impact an individual’s health and constitute a high socioeconomic burden (3–5).When pain persists for at least 3 months, it is classified as chronic (6). Management of chronic low back pain and chronic neck pain mainly consists of conservative treatment options, i.e., pain neuroscience education, exercise therapy, physical activity, acupuncture, pharmacotherapy, or multidisciplinary rehabilitation (5, 7, 8). However, interventional treatment options, among which are epidural injections, disc decompression, nerve blocks, or intradiscal procedures, may be indicated in chronic or refractory spinal pain syndromes (9, 10). Previous research indicates that in 10–40% of spinal surgeries, depending on the exact type of surgery, pain reoccurs or persists leading to the so-called Persistent Spinal Pain Syndrome Type II (PSPS-T2) (11).

PSPS-T2 patients are considered a heterogeneous group based on diversified aetiologies and have multilevel complaints (12). Besides pain and functional problems, these patients report a high work disability that leads to reduced work effectiveness, changes in job responsibilities, and unemployment rates of 50.9–81.5% (11, 13–17). Work participation, defined as the capability and/or opportunity to participate in the workforce and fulfil one’s work role, is consequently reduced for these patients (18). Being able to return to working activities after a period of sick leave or unemployment, otherwise known as return to work (RTW), is a common measure of work participation (18, 19). Longer periods of limited work participation due to long-term unemployment, inability to, or delayed RTW, affect a patient’s ability to maintain an independent lifestyle, impact quality of life, and simultaneously impose a high economic burden to society due to indirect costs (20–23). The literature reports that 61–87% of PSPS-T2 patients are within the working age range (24, 25). This, in combination with the high unemployment rates, emphasizes the need for improvements in work participation as realistic and major treatment goals (26). Management of PSPS-T2 aims to reduce pain and improve functioning and has been the subject of considerable research. Spinal cord stimulation, rehabilitation, psychological therapy, and minimal invasive procedures are considered to be the most effective, while poor evidence is presented for the efficacy of pharmacological therapies and reoperations (27–31). Despite the fact that optimal medical management is effective to decrease pain and improve disability, PSPS-T2 patients do not achieve successful work participation (15, 27–31). Although there is strong evidence that working can reverse the long-term negative effects of unemployment on health and well-being, detailed studies on interventions for work participation and their efficacy are currently lacking (20, 23, 32–34). It should thus be imperative to search for those interventions or therapies that are most effective to facilitate work participation.

To our knowledge, a review on nonsurgical, nonpharmacological, multidisciplinary rehabilitation to improve work participation (e.g., job coaching, ergonomics, pain management, vocational therapy, etc.) for PSPS-T2 patients has not yet been conducted. This systematic review therefore presents an up-to-date overview of the current body of literature on rehabilitation interventions to improve work participation, and their effectiveness for PSPS-T2 patients.

## METHODS

This systematic review adheres to the Preferred Reporting Items for Systematic Review and Meta-Analysis (PRISMA) statement recommendations (35). The protocol was prospectively registered in the PROSPERO database (CRD42022346091).

### Search strategy and eligibility criteria

The search strategy was developed according to the PICO (Population–Intervention–Comparison(s)–Outcome) framework (36). The population was defined as chronic spinal pain patients with previous spinal surgery, the intervention as nonsurgical, nonpharmacological rehabilitation and the outcome as work participation. The intervention could be compared to standard care, no intervention, or any other type of intervention.

MEDLINE (via PubMed), Embase, Scopus, and Web of Science were searched from inception to 1 September 2023 to identify potentially relevant studies. Additionally, reference lists were checked and citation tracking was performed to identify all relevant studies (36). The complete search strategy for each database is detailed in Table SI.

All studies were screened against predetermined inclusion criteria as presented in Table I. The following inclusion criteria were used: (*i*) experimental, quasi-experimental, and observational studies with and without a control group; (*ii*) population including adults (≥ 18 years) with a history of at least 1 spinal surgery, currently experiencing chronic back and/or neck pain (≥ 3 months); (*iii*) nonsurgical, noninvasive, nonpharmacological rehabilitation intervention(s) or rehabilitation programme(s); (*iv*) work-related outcome(s) relating to work participation (e.g., RTW, time to RTW, work-capacity score(s), sick leave, absenteeism, employment status, work disability, etc.); (*v*) in English, French, Dutch, and German languages. Studies enrolling participants receiving any type of pharmacological/surgical therapy as an intervention were exluded. Finally, studies that were available only in abstract format were excluded.

**Table I:** Inclusion and exclusion criteria for screening

Inclusion	Exclusion
Experimental, quasi-experimental and observational studies with and without a control group	Reviews, meta-analyses, case reports, abstracts from conferences, proceedings, expert opinions, letters to the editor, guidelines
English, French, Dutch, German	Other languages than English, French, Dutch, German
Chronic pain (≥ 3 months), back/neck pain, minimum 1 spinal surgery	Subacute or acute pain (< 3 months), non-back/-neck pain, no previous surgery
Human	Non-human
Aged ≥ 18	Aged < 18
Nonsurgical, nonpharmacological rehabilitation interventions, rehabilitation programmes, e.g., acupuncture, TENS, radiotherapy, virtual reality, advice, hypnosis etc.	Medical and pharmacological interventions, e.g., epidural injection, nerve block, fusion surgery, decompression, discectomy, telemedicine, pharmaco-intervention
Work-related outcomes, e.g., work-ability, (un)paid employment, sick leave, return to work, absenteeism, work performance etc.	Non-work-related outcomes

TENS: Transcutaneous electrical nerve stimulation.

In the case of a mixed population of chronic spinal pain patients with and without a history of spinal surgery, a study was considered and study results on the spinal surgery history subgroup were extracted whenever possible. If a study contained missing outcomes of interest, the authors were contacted.

The employment status of participants after any rehabilitation intervention was used as the primary outcome parameter. Secondary outcomes included time between the end of rehabilitation and start of return to employment, absenteeism, sick leave, work-ability scores, and work disability.

### Data extraction and analysis

All search results were exported to EndNote (EndNote v. X9, Thomson Reuters, San Francisco, CA, USA), where duplicates were removed. The title and abstract of the unique results were independently reviewed by 2 reviewers on the inclusion and exclusion criteria using Rayyan software (Rayyan Systems Inc; https://www.rayyan.ai/) (37). Full-text publications of potentially relevant references were obtained and independently assessed by 2 reviewers. Relevant data from included studies were extracted by 1 reviewer and verified by a second using an *a-priori* developed data extraction form comprising first author, publication year, study design, population, intervention, comparator/co-intervention, work-related outcomes, and summary of results. The work-related outcomes were presented as originally reported, without any conversion to a standardized measure. Any discrepancies in the initial or full text screening and data extraction were addressed during a consensus meeting with both reviewers and decided by a third reviewer. The methodological quality of the retained publications was independently assessed using the modified Downs and Black quality assessment checklist (Table SII). The Downs and Black checklist was designed for evaluation of both randomized and non-randomized comparative studies (38). Studies are scored on 27 items concerning reporting, external validity, internal validity (confounding), and power. This implies that the maximal scores for randomized, non-randomized, and non-controlled studies are respectively 28, 25, or 20. The following suggested cut-off scores were used: “Excellent” (26–28); “Good” (20–25); “Fair” (15–19); and “Poor” (≤ 14) (39). To avoid the selective reporting of study findings, studies were not excluded based on the results of the quality and risk of bias assessment. The GRADE methodology was used to assess the certainty of evidence (40).

## RESULTS

### Study selection

**Fig. 1:**
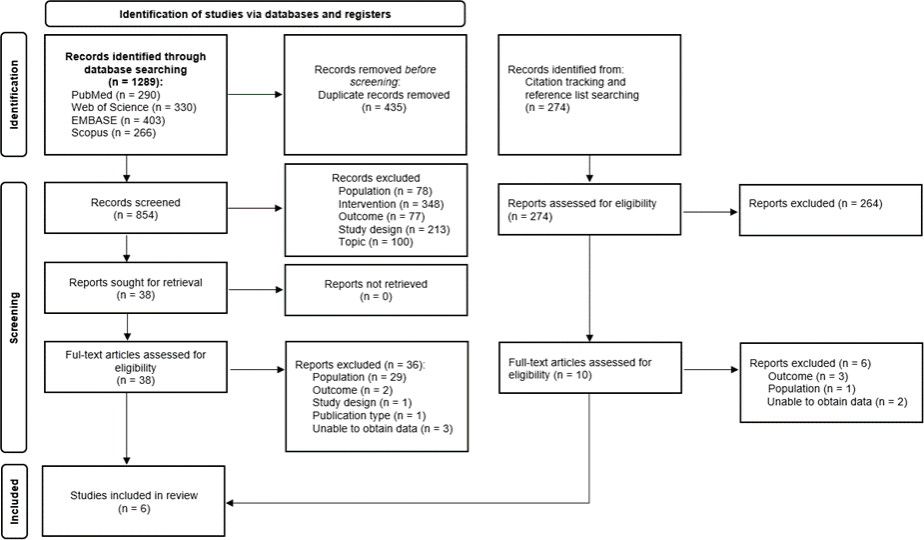
Preferred Reporting Items for Systematic Review and Meta-Analysis (PRISMA) study flow diagram.

The search yielded a total of 1,289 potentially relevant records: 403 for Embase, 330 for Web of Science, 290 for PubMed, and 266 for Scopus. A detailed overview of the literature search is provided in Fig. 1.

After removal of duplicates (*n* = 435), 854 unique records remained. Subsequently, title and abstract were screened, leading to the exclusion of 816 records. The main reasons for exclusion were (a) not evaluating a rehabilitation intervention (*n* = 348); (b) not evaluating work-related outcomes (*n* = 77); (c) study population not consisting of chronic spinal pain patients with previous spinal surgery (*n* = 78). Other studies were excluded due to study design, publication type, language, or topic (*n* = 313).

After exclusion, 38 articles were retrieved for full-text evaluation. Citation tracking and reference list searching revealed another 274 potential records and resulted in an additional 10 articles for full-text evaluation. Thus, 48 publications were comprehensively screened in full, whereafter 6 publications were retrieved for inclusion in this systematic review (41–46). For 5 potential records with a mixed population of patients with and without PSPS-T2, the authors were contacted. This led to the exclusion of all 5 records due to not being able to obtain work-related outcome results on the PSPS-T2 subgroup. Only a narrative synthesis was performed due to the limited number of included studies and presence of between-study heterogeneity, which precluded grouping of studies and meta-analysis.

### Study characteristics

The characteristics for the included studies are detailed in Table II. The included studies were published between 1994 and 2023. Three studies were conducted in France, 2 in the USA and 1 in Switzerland. One randomized controlled trial (RCT) (45), 2 retrospective case series (41, 44), 1 prospective case series (43) and 2 prospective cohort studies (42, 46) were included. All studies recruited a mixed population including both participants with and without PSPS-T2. The total number of PSPS-T2 patients ranged between 18 and 119, totalling 240 patients who followed rehabilitation. In each study, the PSPS-T2 subgroup was a minority compared with the total study sample, comprising 10–30% of all participants. The mean age ranged between 40 and 43.8 years, and study populations were mostly male dominated.

**Table II:** Characteristics of included studies.

Study	Population, *n* (amount of patients with a previous surgery (i.e., PSPS-T2 patients))	Intervention	Comparators	Outcomes
Porteau-Cassard et al., retrospective case-series (41)	144 (18), mean age 43.8 ± 9.7; sex 50% male.	Back school programme (physical therapy, material handling techniques and occupational therapy) 5-day time period Providing team of rheumatologist, occupational therapist, manutention expert, physiotherapists and dietitian	No comparators	Days off work (mean+SD): At baseline: 78.5 ± 82.4 At 6-month follow-up: 36±75.8
Burke et al., prospective cohort study (42)	397 (119), mean age 36 (T), 37 (C); sex 78% male (T), 81% male (C)	Functional restoration programme (strength testing, educational programme and work simulation) Minimum of 1 week time period Providing team not specified	Comparator group received no treatment	RTW rate: At 6-month follow-up: 54% (T), 32% (C) At 12-month follow-up: 72% (T), 14% (C)
Poulain et al., prospective case series (43)	105 (35), median age 44; sex 45% male	Functional restoration programme (physical exercise, relaxation, education and CBT) 4-week time period, 5 days a week, 6 h a day Providing team of physician, psychologist, physiotherapists, ergonomist, social worker and dietitian	No comparators.	RTW: Prior back surgery is a non-significant variable predicting long-term RTW after intervention (*p* > 0.1)
Tavares-Figueiredo et al., retrospective case series (44)	99 (15), mean age 40.8 ± 10.0; sex 63.6% male	Validated self-care programme (physical and educational approaches) 3-week time period, 5 days a week, 8 h a day Providing team of physical and occupational medicine physician, rehabilitation physician, rheumatologist, social worker, physical and occupational therapists, nurses, psychologist, dietitian and pain physician	No comparators	Work status (proportion of participants working/not working): At baseline: 5/10 At 6-month follow-up: 4/4 (MD = 7) At 12-month follow-up: 3/3 (MD = 9)
Cui et al., randomized controlled trial (45).	140 (15), median age 50.5 (T), 54.5 (C); sex 32% male 74.3% (T), 64.3% (C) within working age *	Tailored digital care programme (exercise, education and CBT) 8-week time period, 3 days a week, 20 min a day Providing team of physical therapists	Comparator group received evidence-based in-person physiotherapy	Work status (proportion of participants working/not working): At baseline: 4/5 (T), 4/2 (C) At 8-week follow-up: 2/3 (T), 2/1 (C) (MD = 7)
Ibrahim et al., prospective cohort study (46)	201 (38), mean age 40.0; sex 59% male	Multidisciplinary biopsychosocial rehabilitation programme 4-week time period, 5 days a week, 100 h total Providing team of rheumatologist, rehabilitation physician, pain specialist, psychiatrist, physiotherapists, occupational therapists and psychologist	No comparators	Work status (proportion of participants working/not working): At baseline: 9/27 (MD = 2) At programme end: 10/23 (MD = 5) At 6-month follow-up: 11/13 (MD = 14) At 18-month follow-up: 13/7 (MD = 18)

T: treatment group, C: comparator group, RTW: return to work, CBT: cognitive-behavioural therapy, MD: missing data.

* Data reported for the entire study population.

All 6 studies described an intervention that combined a physical and educational component. Most studies included a diagnostic evaluation as the starting point of the intervention (42, 44–46). The studies by Poulain et al. and Ibrahim et al. were the only to include a management component (43, 46). A work-related component was described in 2 studies; Burke et al. included a work-simulation, and Ibrahim et al. specified workplace adaptations and workplace visits (42, 46). All studies, except 1, individually adapted specific components of the intervention programme to address patient capacity (45, 46), goals (41, 46), or work situation (41–43, 46). The study by Tavares-Figueiredo et al. (44) did not describe any adaptations but used the diagnostic evaluation to establish individual therapy objectives.

Four studies described inpatient rehabilitation programmes, whereas only 1 study examined an outpatient programme (41–44, 46). The RCT by Cui et al. (45), on the other hand, compared inpatient rehabilitation with an outpatient digital programme. The length of the different interventions ranged from 1 to 8 weeks, from 20 min per day to 8 hours per day, and from 2 to 5 days a week. The study of Ibrahim et al. additionally included a refresher course at 6 months following completion of the intervention.

In most studies, a multidisciplinary team provided the rehabilitation, composed of both physicians and paramedical healthcare professionals, namely physiotherapists, occupational therapists, dietitians, social workers, psychologists, or nurses (41–44, 46). The study by Cui et al. described a monodisciplinary team of physical therapists (45).

Only 2 studies included a comparator group. The study by Burke et al. compared rehabilitation with no treatment, and the RCT by Cui et al. described conventional physiotherapy as comparator (42, 45). The other included studies were cohort studies and case series lacking a control group.

### Risk of bias and quality

The methodological quality scores based on the modified Downs and Black checklist are presented in Table III. The observational studies by Porteau-Cassard et al. (41), Poulain et al. (43), and Tavares-Figueiredo et al. (46) were scored as “poor” (respectively 12, 13, and 12/28) with considerable risk of bias towards sampling, loss to follow-up and missing data. The studies by Burke et al. (42) and Ibrahim et al. were scored as “fair” (respectively 16 and 17/28), and the RCT by Cui et al. (45) was scored as “good” (24/28), although risk of bias toward blinding is present.

**Table III:** Summary of methodological screening (modified Downs and Black checklist).

	Reporting										External validity			Internal validity													Power	
	Hypothesis	Main outcomes	Participant characteristics	Interventions	Distributions of principal confounders	Main findings	Estimates of the random variability	Adverse events	Characteristics of participants LTFU	Actual probability values reporting	Representativeness of participants asked	Representativeness of included participants	Representativeness of treatment accommodation	Blinding of study participants	Blinding of assessors	Data dredging	Adjustment for different follow-up duration	Appropriateness of the statistical tests	Compliance with the intervention reliable	Main outcome measures valid and reliable	Participants recruited from the same population	Study participants recruited over same period of time	Study participants randomized to intervention groups	Randomized to intervention assignment concealed	Adjustment for confounding	Losses of participants missing data taken into account	Power	Total
**Porteau-Cassard (41)**	1	1	1	0	1	1	1	0	1	1	0	0	1	0	0	0	0	1	1	0	0	0	0	0	0	1	0	12
**Burke (42)**	1	1	0	1	2	1	1	0	1	1	0	0	1	0	0	0	1	1	1	1	1	1	0	0	0	0	0	16
**Poulain (43)**	1	1	1	1	1	1	1	0	1	1	0	0	1	0	0	0	0	1	1	1	0	0	0	0	0	0	0	13
**Tavares-Figueiredo (44)**	1	1	1	1	1	1	1	0	1	1	0	0	1	0	0	0	0	1	1	0	0	0	0	0	0	0	0	12
**Cui (45)**	1	1	1	1	2	1	1	1	1	1	1	0	1	0	0	1	1	1	1	1	1	1	1	0	1	1	1	24
**Ibrahim (46)**	1	1	1	1	2	1	1	0	1	1	0	0	1	0	0	1	1	1	1	1	0	0	0	0	0	1	0	17

LTFU: lost to follow-up.

The level of evidence for the included studies was downgraded due to concerns regarding indirectness for all studies, the lack of blinding for the RCT by Cui et al., and additionally due to risk of selection bias and missing data/loss to follow-up in the observational studies (Table SIII).

### Work-related outcomes

Four out of 6 studies reported on the employment status both before and after the intervention by providing the absolute number of working participants with PSPS-T2 or a RTW rate (42, 44, 45). In the study by Burke et al., PSPS-T2 patients receiving rehabilitation were compared with PSPS-T2 patients not receiving any treatment (42). Rehabilitation led to a higher amount of PSPS-T2 patients who achieved successful RTW at 6 months (54% vs 32%) and at 1 year (72% vs 14%) (42). Patients with a previous spinal fusion were more than 5 times as likely to achieve RTW if they received rehabilitation compared with no rehabilitation at 1 year (*p =* 0.004) (42). Patients with a previous laminectomy who followed rehabilitation were 1.5 more likely to return to work after 6 months compared with those without rehabilitation (42). Ibrahim et al. reported on an increase in the work rate of participants after rehabilitation immediately following the programme, and at 6-month and 18-month follow-up (missing data *n* = 18) (46). Likewise, in the study by Tavares-Figueiredo et al. there was an increase in the percentage of participants at work after rehabilitation at 6-month and 12-month follow-up (missing data *n* = 9) (44). Cui et al. (45) reported on a total of 15 participants; however, in both the intervention and control group there was no change in employment status before vs after rehabilitation (missing data *n* = 7). In the case-series by Poulain et al., having prior spinal surgery was reported as a non-significant variable in predicting long-term RTW after rehabilitation (*p* > 0.1) (43). The case-series by Porteau-Cassard et al. was the only study to present data on sick leave and reported a non-significant difference in the number of days off work at 6 months after the intervention, compared with baseline (78.5 ± 82.4 to 36 ± 5.8), which did not change between the 6-month and 12-month follow-up (41).

## DISCUSSION

This systematic review provides an overview of the literature on rehabilitation therapy to improve work participation, and its effectiveness in PSPS-T2 patients. An extensive search in well-known databases resulted in 6 relevant studies. Our findings suggest that nonsurgical, nonpharmacological rehabilitation may increase work participation, but the evidence is uncertain.

Despite this uncertainty, there are important take-home messages from our results that can influence daily clinical practice. The included studies are in line with previous research in chronic pain populations, stating that multidisciplinary and multicomponent interventions are more effective than monodisciplinary and single-component counterparts for work-related outcomes, especially in patients who failed to show improvements after surgical interventions (47–49). This could explain why the study by Cui et al. (45), investigating a monodisciplinary intervention, presents negative results, while the other studies indicate positive effects (41–44, 46). Furthermore, therapy intensity (i.e., amount of hours of therapy per day) in the study by Cui et al. is significantly lower compared with the other programmes (20 to 30 min per day, 1 day per week) (45). However, the medical literature remains inconclusive on the intensity and duration of rehabilitation needed to achieve the best effects (50, 51). Similarly, there is no clear consensus on whether group-based or individual rehabilitation is more effective, whether in- or outpatient rehabilitation is preferable, and whether in-person or digital rehabilitation is more beneficial (52). This might highlight the relevance of a programme tailored to a patient’s preferences, needs, and abilities, described in all the included studies in our study (by, e.g., adaptations of exercises, addressing patient-specific shortcomings, personal and meaningful goalsetting, etc.). Research on work participation supports this personalized approach as it minimizes the risk that essential personal, social, or work-related information will be bypassed (26, 53, 54). On the other hand, evidence on personalized rehabilitation to improve work participation compared with usual care or standard treatment is still scarce (52, 55). Surprisingly, only 4 studies include a diagnostic or assessment component and just 2 studies have a management component, both of which are essential to tailor to patient preferences and abilities (41, 43).

The perspective on work disability and work participation has evolved from a narrow physical focus towards a more comprehensive biopsychosocial framework (56). Over the past decade, there has been a notable increase in the recognition of this framework (56–58). This has led to more evidence suggesting that rehabilitation for socioeconomic outcomes should be multidisciplinary and encompass the biological, psychological, and social aspects of a patient’s ability to work (56–58). The included interventions, although multidisciplinary in nature, differ from recommendations described in models stemming from the biopsychosocial framework (e.g., Sherbrooke model and ecological case management model) (56, 57, 59). It should be noted that the publication dates of the included studies range from 1994 to 2023. Therefore, it should be acknowledged that not all studies could have implemented the most recent recommendations. The interventions in each included study used 2 basic therapeutic modalities: a physical exercise component and an educational component (41–46). These components are generally considered cornerstones of rehabilitation programmes (60). In and of themselves, these components do not improve work participation (61, 62). The physical ability to perform work does not guarantee RTW and informing patients on job adaptations or self-management strategies does not effectively reduce absenteeism (61, 62). These 2 components merely represent the first step in the biopsychosocial perspective. A number of other aspects are critical for work participation. Identifying personal, social, and work-related factors (i.e., job satisfaction, worker perception, recovery expectations, self-efficacy, readiness, etc.), analysing the work-disability situation, actively involving the employer, setting and adjusting individualized objectives, considering work modifications, workplace involvement, ensuring interdisciplinary coordination, and personaliszation are necessary components for rehabilitation directed at improving work participation (63–67). Interestingly in this review, an active work-related component is described in only 2 studies. Burke et al. described a work simulation component, and Ibrahim et al. investigated advice for workplace adaptations and workplace visits, resulting in higher RTW rate and improved work rate after intervention (42, 46). Prior research and theoretical frameworks have identified active work-related components (i.e., workplace interventions, targeted vocational rehabilitation) as effective and essential to improve work participation among adults with chronic physical conditions, including chronic low back pain (20, 32, 56, 57, 59, 68–72).

Recent advances in healthcare such as novel imaging techniques (i.e., MR neurography, or SPECT/CT), the growth of minimally invasive surgical approaches, and the integration of emerging technologies (i.e., image-guided or robot-assisted surgery, virtual reality) have led to increased safety, faster recovery, and improved clinical outcomes after spinal surgery (73, 74). Despite this, and guidelines addressing the overuse of surgery in low back pain, the recent prevalence of PSPS-T2 remains high at 14.97% (75). The low to very low certainty of the evidence identified in this review, together with the fact that patients with a history of surgery are often excluded from trials on chronic low back pain, highlights the importance of high-quality clinical trials that evaluate rehabilitation for work participation in a large sample of PSPS-T2 patients (76). As these patients are more likely to incur substantial medical costs, there is a significant potential for socioeconomic improvement. Our results are a first step and a clear call for future studies to continue comprehensive research on how to increase the percentage of work participation, thereby reducing the risk of wasting scarce resources on interventions that have little effect on work participation. Both research and daily clinical practice would greatly benefit from a more transparent conceptualization of biopsychosocial rehabilitation for work-related outcomes, such as work participation. The authors advocate the use of established frameworks (i.e., biopsychosocial framework) and models (i.e., Sherbrooke model) as the foundation to guide the content and intensity of rehabilitation and recommend including active work-related intervention components. Moreover, a single outcome measure is unlikely to cover the broad concept of work participation. The authors therefore advocate using at least both a time-based (i.e., time to RTW) and status-based measure (i.e., work status or RTW status). A recent concept analysis of biopsychosocial rehabilitation for chronic low back pain in the working population identified personalization as one of the key attributes (77). Along with the results of this review, the authors recommend a personalized approach based on proper assessment when it comes to rehabilitation for work participation. Finally, considering the growth of individualized medicine, the inherent complexity of individualized approaches in chronic pain, and the findings of this review, follow-up research was initiated. A clinical trial investigating the effects of personalized biopsychosocial rehabilitation targeting RTW for PSPS-T2 patients implanted with SCS is currently ongoing (78).

### Strengths and weaknesses

This systematic literature review is the first to summarize the available evidence on the effectiveness of rehabilitation to improve work participation in PSPS-T2 patients. A major strength is the predetermined systematic methodology, which ensured a comprehensive examination of the literature, while simultaneously minimizing risk of bias. An unrestricted search strategy in terms of study design, publication date, and language, as well as citation tracking, and reference list searching add to the strengths. However, this review also has some limitations. First, PSPS-T2 patients are more likely to have an extended medical treatment history and sustained periods of prolonged absence from employment, which makes finding successful treatments arduous (79, 80). In addition, a significant proportion of the literature on chronic spinal pain patients has a strong medical focus, resulting in surgical, pharmacological, or other invasive interventions (29). Therefore, the number of publications on rehabilitation for work-related outcomes in PSPS-T2 patients is potentially limited and could explain why only a small number of studies of mostly poor to fair quality and small sample size were identified. A considerable number of studies described a history of neck and/or back surgery as an exclusion criterion for patient recruitment and therefore had to be excluded from this review. This once more highlights the importance of this review and warrants the need for future research to comprehensively investigate this population, despite its potential perceived obstacles. The limited number of included studies, issues on sample size and missing data, as well as clinical heterogeneity, resulted in a downgrade of the certainty of evidence according to GRADE and precluded a meta-analysis, potentially limiting generalizability. Lastly, following full-text screening, several studies (*n* = 5) were excluded as the authors were unable to provide results on the work-related outcomes of the subgroup of PSPS-T2 patients as part of their included study population. However, it is uncertain whether the addition of these studies would have resulted in a non-ambiguous conclusion, based on high-quality evidence.

### Conclusion

The current medical literature lacks evidence to provide recommendations regarding the effectiveness of rehabilitation focusing on work participation in PSPS-T2 patients in the short and long term. Considering the growing prevalence of chronic back pain and the increasing number of back surgeries performed worldwide, future research should continue to investigate rehabilitation options with the aim to improve professional reintegration in PSPS-T2 patients and beyond.

## Supplementary Material


